# The molecular chaperone Hsp90 maintains Golgi organization and vesicular trafficking by regulating microtubule stability

**DOI:** 10.1093/jmcb/mjz093

**Published:** 2019-09-27

**Authors:** Yuan Wu, Yubo Ding, Xiudan Zheng, Kan Liao

**Affiliations:** Key Laboratory of Systems Biology, CAS Center for Excellence in Molecular Cell Science, Shanghai Institute of Biochemistry and Cell Biology, Chinese Academy of Sciences, University of Chinese Academy of Sciences, Shanghai 200031, China

**Keywords:** Hsp90, microtubule, Golgi fragmentation, vesicular trafficking, MAP4

## Abstract

Hsp90 is an abundant and special molecular chaperone considered to be the regulator of many transcription factors and signaling kinases. Its high abundance is indicative of its involvement in some more fundamental processes. In this study, we provide evidence that Hsp90 is required for microtubule stabilization, Golgi organization, and vesicular trafficking. We showed that Hsp90 is bound to microtubule-associated protein 4 (MAP4), which is essential for maintaining microtubule acetylation and stabilization. Hsp90 depletion led to the decrease in MAP4, causing microtubule deacetylation and destabilization. Furthermore, in Hsp90-depleted cells, the Golgi apparatus was fragmented and anterograde vesicle trafficking was impaired, with phenotypes similar to those induced by silencing MAP4. These disruptive effects of Hsp90 depletion could be rescued by the expression of exogenous MAP4 or the treatment of trichostatin A that increases microtubule acetylation as well as stability. Thus, microtubule stability is an essential cellular event regulated by Hsp90.

## Introduction

The 90-kDa heat shock protein (Hsp90) is a highly abundant and conserved molecular chaperone with ubiquitous distribution in eukaryotic cells (Craig et al., 1993; Feder and Hofmann, 1999). In mammalian cells, Hsp90 is composed of two isoforms: the inducible Hsp90α and the constitutive Hsp90β (Sreedhar et al., 2004). These two isoforms are highly homologous with 86% amino acid sequence identity, and each of the isoforms could functionally compensate for the other (Farrelly and Finkelstein, 1984; Bardwell and Craig, 1987; McDowell et al., 2009).

The binding of Hsp90 to its client proteins stabilizes or activates them by facilitating their protein-folding and conformational change (Pratt and Toft, 2003; Pearl and Prodromou, 2006; Wandinger et al., 2008). Since many Hsp90 client proteins are signaling kinases and transcription factors, most studies about Hsp90 have been focused on its functions in signal transduction (Miyata and Yahara, 1992; Xu and Lindquist, 1993; Blagosklonny et al., 1996; Fontana et al., 2002; Picard, 2002). In unstressed cells, Hsp90 accounts for 1%–2% of total cytoplasmic proteins (Csermely et al., 1998). Its high abundance in the cell is indicative of its involvement in other cellular activities.

There are reports that inhibition of Hsp90 by Hsp90 inhibitors causes photoreceptor degeneration in rat, beagle dog, and human (Pacey et al., 2011; Zhou et al., 2013; Kanamaru et al., 2014). During our previous study of Hsp90α-deficient mice, we found that Hsp90α is the major Hsp90 isoform in retina and its deficiency caused retinitis pigmentosa (RP) (Wu et al., 2020). RP is a common inherited retinal disease characterized by gradual photoreceptor degeneration and eventual blindness (Dryja and Li, 1995; Rivolta et al., 2002). Further investigations revealed that Hsp90α deficiency induced Golgi disintegration and rhodopsin mislocalization in photoreceptors. The Golgi apparatus is the membrane system in which post-translational protein modification, maturation, and transport take place. Rhodopsin is synthesized in the inner segment of the photoreceptor and then transported to the membrane discs in the outer segment. It requires the Golgi apparatus for this transport process. The Golgi apparatus disintegration in Hsp90α-deficient photoreceptors caused rhodopsin mislocalization, impaired intersegment protein transport for cellular turnover, and induced photoreceptor degeneration. Based on these results, we postulate that Hsp90 plays an essential role in vesicular membrane trafficking.

To ascertain the mechanism by which Hsp90 deficiency impairs cellular vesicle trafficking, the temperature-sensitive vesicular stomatitis virus glycoprotein (VSVG^tsO45^) transport system was used to provide a signal to follow the vesicle trafficking in the cell (Gallione and Rose, 1985; Gougeon et al., 2002). Tracking VSVG^tsO45^ transport in Hsp90-depleted HeLa cells could reveal the effect of Golgi disintegration on cellular vesicle trafficking. In our current study, we found that Hsp90 depletion caused a delay of VSVG^tsO45^ in reaching the plasma membrane (PM). It could leave the endoplasmic reticulum (ER), but was retained in disintegrated Golgi. This impaired anterograde vesicle trafficking could be restored by trichostatin A (TSA) treatment or exogenous expression of microtubule-associated protein 4 (MAP4), a microtubule-associated protein that interacts with Hsp90. Taken together, our results provided strong evidence that Hsp90 deficiency induced microtubule destabilization at least in part by promoting MAP4 degradation and microtubule deacetylation, which caused Golgi fragmentation and impaired vesicular trafficking.

## Results

### The anterograde vesicle transport impaired by loss of Hsp90

**Figure 1:**
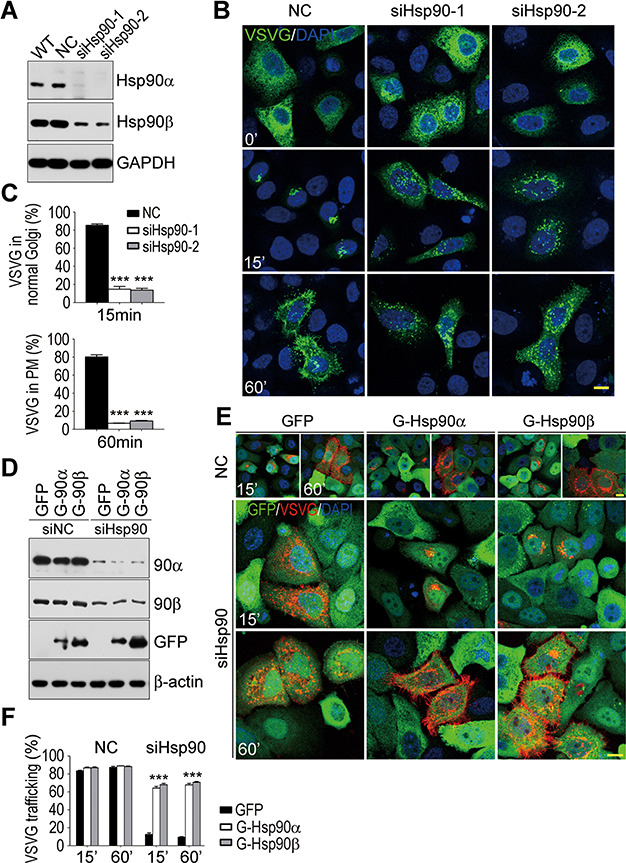
Blocked anterograde vesicle trafficking in Hsp90-depleted HeLa cells. (**A**) Knockdown of Hsp90 by RNAi in HeLa cells. WT, wild-type HeLa cells; NC, negative control cells transfected with a scrambled siRNA sequence; siHsp90-1 and siHsp90-2, HeLa cells transfected with two different siRNA sequences targeting both isoforms of Hsp90. GAPDH is the protein loading control. (**B**) Blocked VSVG^tsO45^-GFP trafficking in Hsp90-depleted HeLa cells. Control or Hsp90-depleted cells expressing VSVG^tsO45^-GFP were shifted from 40.5°C to 32°C. 0′, 15′, and 60′ indicate the incubation time at 32°C. (**C**) Quantification of VSVG^tsO45^-GFP localization in control or Hsp90-depleted cells. Cells with VSVG^tsO45^-GFP in the tightly compacted Golgi (15′) or the PM (60′) were measured, and their percentages in VSVG^tsO45^-GFP-positive cell population were calculated. The results were the average of three independent experiments and at least 150 cells were analyzed per group for each experiment. Error bars represent SEM. Student’s *t*-test, ***P < 0.001. (**D**) Stable expression of siRNA-resistant GFP-Hsp90α or GFP-Hsp90β isoform in the presence of Hsp90 siRNA. Stable HeLa cells expressing GFP or siRNA-resistant GFP-Hsp90α or GFP-Hsp90β were treated with siRNA oligomers. Endogenous Hsp90α or Hsp90β was detected with anti-Hsp90α or anti-Hsp90β antibody, and exogenous GFP-tagged Hsp90α or Hsp90β was detected with anti-GFP antibody. (**E**) Restoration of VSVG^tsO45^ transport by exogenous GFP-Hsp90α or GFP-Hsp90β supplement in Hsp90-depleted cells. (**F**) Quantification of VSVG^tsO45^-Mcherry localization in Hsp90-depleted cells with siRNA-resistant Hsp90 supplement. The measurement and calculation were the same as those in **C**. The results were the average of three independent experiments, and at least 125 cells were analyzed per group for each experiment. Error bars represent SEM. Student’s *t*-test, ***P < 0.001. Scale bar, 10 μm.

Two different siRNAs (siHsp90-1 and siHsp90-2) were used to deplete Hsp90 in HeLa cells. As shown in Figure 1A, both Hsp90α and Hsp90β were successfully knocked down by these two siRNAs. VSVG is a vesicular stomatitis virus glycoprotein synthesized in the ER and transported through the Golgi to the PM. Its temperature-sensitive mutant, VSVG^tsO45^, is retained in the ER at 40.5°C due to misfolding. After shifting to 32°C, the misfolded VSVG^tsO45^ protein in the ER will refold properly and enter the protein trafficking system. The travelling of VSVG^tsO45^ in cellular membrane system represents the anterograde vesicle trafficking. To evaluate the effect of Hsp90 deficiency on cellular anterograde vesicle trafficking, a GFP-tagged VSVG^tsO45^ was expressed in Hsp90-depleted HeLa cells at 40.5°C and then its trafficking was traced at 32°C. After lowering the temperature to 32°C, VSVG^tsO45^-GFP in most control cells was transported to the tightly compacted Golgi apparatus at 15 min and reached the PM at 60 min (Figure 1B). However, in Hsp90-depleted cells, VSVG^tsO45^-GFP was distributed in fragmented patches at 15 min and remained the same at 60 min (Figure 1B and C). Barely any VSVG^tsO45^-GFP reached the PM within 60 min after shifting the temperature from 40.5°C to 32°C in Hsp90-depleted cells. The protein trafficking was apparently impaired in cells without Hsp90. In addition, at the permissive temperature VSVG^tsO45^-GFP could leave the ER in time, but could not form the typical Golgi morphology (Figure 1B).

To ascertain that it was the loss of Hsp90 that impaired the anterograde vesicle trafficking, we performed rescue experiments by transfecting Hsp90 siRNAs and VSVG^tsO45^-Mcherry into HeLa cells expressing siRNA-resistant GFP-Hsp90α or GFP-Hsp90β protein. The siRNA-resistant Hsp90 constructs were generated by mutating the sites that matched the Hsp90 siRNAs without altering their protein sequences. Thus, the siRNA-resistant Hsp90 proteins could exist stably in the presence of the Hsp90 siRNAs (Figure 1D). The supplement of exogenous GFP-tagged Hsp90α or Hsp90β could efficiently restore the inhibited VSVG transport (Figure 1E and F). At 32°C, VSVG^tsO45^-Mcherry reached the tightly compacted Golgi within 15 min and the PM within 60 min in most Hsp90-depleted HeLa cells with exogenous GFP-tagged Hsp90α or Hsp90β, but not with exogenous GFP. The possibility of off-target effects by Hsp90 siRNAs to inhibit vesicle transport was ruled out.

### Golgi fragmentation and Rab8 mislocalization in Hsp90-depleted cells

**Figure 2:**
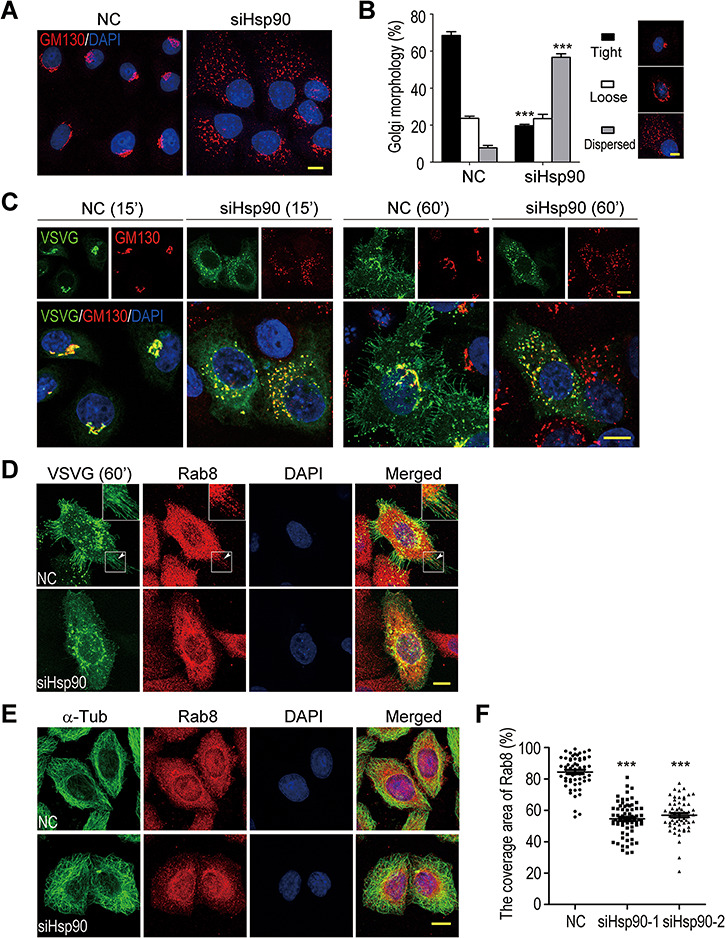
Golgi fragmentation and Rab8 mislocalization in Hsp90-depleted cells. (**A**) The Golgi of Hsp90-depleted HeLa cells. Cells were stained with anti-GM130 antibody to show the Golgi apparatus. (**B**) Quantification of the Golgi with various morphologies in control and Hsp90-depleted cells. Golgi morphologies were categorized into tight, loose, and dispersed types. The results were the average of three independent experiments, and at least 500 cells were analyzed per group for each experiment. Error bars represent SEM. Student’s *t*-test, ***P < 0.001. (**C**) Retention of VSVG^tsO45^-GFP in fragmented Golgi patches of Hsp90-depleted cells at the permissive 32°C after 15 and 60 min. GM130 was stained to show the Golgi. (**D**) Rab8 in cytoplasm of control and Hsp90-depleted cells. Cells were shifted to the permissive temperature for 60-min incubation (60′). Rab8 and VSVG^tsO45^-GFP were detected by immunofluorescence or GFP fluorescence. The arrow heads indicate Rab8 in the VSVG-localizing plasma membrane protrusions. The boxed area is enlarged to show details. (**E**) Retention of Rab8 in perinuclear region of Hsp90-depleted cells. α-tubulin (α-Tub) was stained to show the cell outline. (**F**) Quantification of cytoplasmic area with Rab8 in control or Hsp90-depleted cells. The results were the ratio of the Rab8-covered area relative to the cell outline area. Data are mean ± SEM of the results obtained from at least 59 cells for each group. Student’s *t*-test, ***P < 0.001. Scale bar, 10 μm.

The trafficking of GFP-tagged VSVG^tsO45^ at 32°C can be used to observe the morphology of the Golgi apparatus (Rogalski and Singer, 1984). At the permissive temperature, it reaches the Golgi apparatus at 15 min and its GFP fluorescence morphologically marks the Golgi. Instead of marking the tightly compacted Golgi in control cells, GFP-tagged VSVG^tsO45^ marked many fragmented patches in Hsp90-depleted cells (Figure 1B). Immunofluorescence staining for GM130, a marker protein for *cis*-Golgi, revealed the highly fragmented Golgi in Hsp90-depleted cells (Figure 2A and B). Most Golgi in control cells was in the tightly compacted form with a small percentage in the loose form, whereas the Golgi in Hsp90-depleted cells was mostly in fragmented patches dispersed in the whole cytoplasmic area.

Hsp90 siRNAs depleted both Hsp90α and Hsp90β in HeLa cells and caused almost complete Golgi fragmentation. In the case of depleting either Hsp90α or Hsp90β, Golgi fragmentation was still observed, but was less severe than the loss of both isoforms (Figure 2A and B; Supplementary Figure S1).

In Hsp90-depleted cells, at the permissive temperature VSVG^tsO45^-GFP in those fragmented patches was overlapped with GM130, indicating that it was localized in the fragmented Golgi (Figure 2C). However, after 60 min, VSVG^tsO45^-GFP still remained in those fragmented Golgi patches. Thus, VSVG^tsO45^-GFP in Hsp90-depleted cells could be transported from the ER to the fragmented Golgi, but not from the fragmented Golgi to the PM. As the Golgi apparatus is the core organelle for protein processing and transport (Farquhar, 1985), its fragmentation disrupted the structural foundation for the Golgi to mediate anterograde vesicle trafficking.

Rab8 is a small GTPase associated with membrane vesicles that carry proteins from the Golgi to the PM and is indispensable for VSVG transport (Huber et al., 1993; Bravo-Cordero et al., 2007). The total Rab8 protein in Hsp90-depleted cells was the same as that in control cells (Supplementary Figure S2A). However, the Rab8 distributed in the peripheral region of the Hsp90-depleted cell was significantly decreased in comparison to that of the control cell (Figure 2D–F). The reduced Rab8 in the peripheral region suggested the reduction of vesicles carrying proteins from the fragmented Golgi to the PM. This observation was consistent with the retention of VSVG^tsO45^-GFP in the fragmented Golgi of Hsp90-depleted cells (Figure 2C).

### Microtubule stabilization by Hsp90

**Figure 3:**
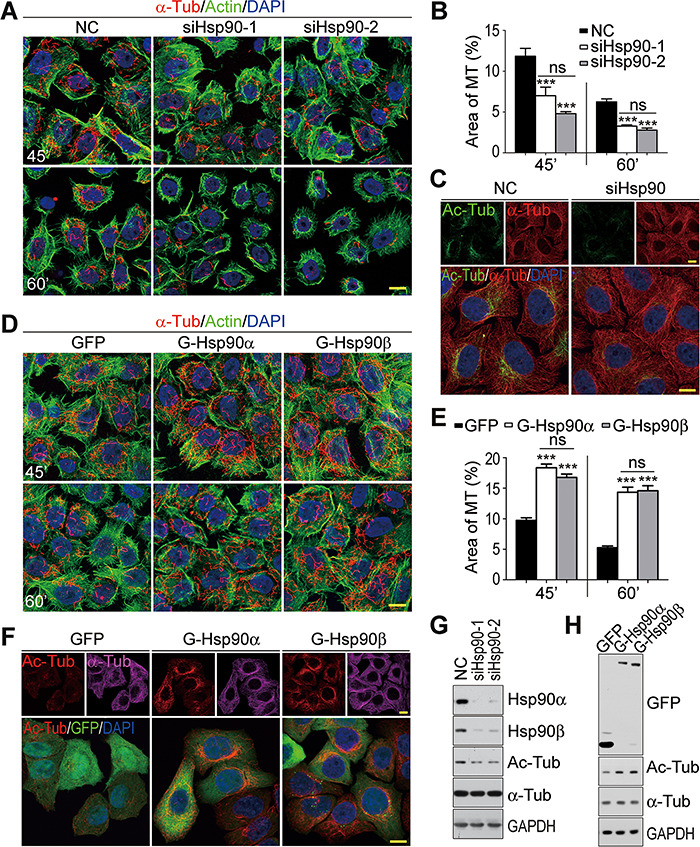
Microtubule destabilization in Hsp90-depleted HeLa cells. (**A**) Microtubule destabilization induced by Hsp90 depletion. Control (NC) and Hsp90-depleted (siHsp90-1 and siHsp90-2) cells were treated with 2 μM nocodazole for 45 or 60 min (45′ or 60′). After extraction and fixation, the cells were stained for α-Tub to show the remaining microtubules and fluorescent-conjugated phalloidin to mark actin and cell outline. (**B**) Quantification of the remaining microtubules (MT) in control or Hsp90-depleted cells after nocodazole treatment. The ratio of microtubule area relative to the cell outline area was calculated to measure microtubule stability. The results were the average of three independent experiments, and 35 cells were measured per group for each experiment. Error bars represent SEM. Student’s *t*-test, ***P < 0.001. (**C** and **G**) Decreased microtubule acetylation in Hsp90-depleted HeLa cells. Microtubule acetylation was detected by immunofluorescence (**C**) and western blot (**G**). Ac-Tub, acetylated α-tubulin. (**D**) Microtubule stabilization induced by Hsp90 overexpression. HeLa cells stably expressing GFP or GFP-tagged Hsp90α or Hsp90β were treated with 2 μM nocodazole for 45 or 60 min and stained for microtubule and actin. (**E**) Quantification of the remaining microtubules after nocodazole treatment in cells overexpressing Hsp90. The results were the average of three independent experiments, and 35 cells were measured for each experiment. Error bars represent SEM. Student’s *t*-test, ***P < 0.001. (**F** and **H**) Increased microtubule acetylation in cells overexpressing Hsp90. Microtubule acetylation was detected by immunofluorescence (**F**) and western blot (**H**). Scale bar, 10 μm.

During the immunofluorescence staining of siRNA-treated HeLa cells for α-tubulin, we observed a possible microtubule network abnormality in Hsp90-depleted cells. In normal cells, the microtubules were uniformly distributed from cell center to periphery. In Hsp90-depleted cells, however, the microtubules were compacted in the cell center and less dense in periphery, suggesting the compromised microtubule stability and organization (Figure 2E). Nocodazole, the microtubule-depolymerizing reagent, was used to analyze microtubule stability (Cambray-Deakin and Burgoyne, 1987; Piperno et al., 1987). After treatment with 2 μM nocodazole for 45 min, there were much more residual microtubules in control cells than in Hsp90-depleted cells (Figure 3A and B). After a 60-min treatment, almost no residual microtubules were present in Hsp90-depleted cells, while substantial amounts of microtubules were still left in control cells. In addition, the overexpression of Hsp90α or Hsp90β in HeLa cells increased the resistance of microtubules to nocodazole (Figure 3D and E). Thus, Hsp90 protein positively regulated microtubule stability.

Microtubules with high-level acetylated α-tubulin are more stable and resistant to depolymerization (Xu et al., 2017). As shown in Figure 3C and G, α-tubulin acetylation was markedly decreased by Hsp90 depletion, a result consistent with the destabilized microtubules in Hsp90-depleted cells (Figure 3A and B). In addition, microtubule stabilization and α-tubulin acetylation were concomitantly increased in cells overexpressing Hsp90α or Hsp90β (Figure 3D–F and H). These results suggested that Hsp90 regulated microtubule stability at least in part through the regulation of α-tubulin acetylation.

### Microtubule destabilization causes aberrant Golgi and vesicle transport in Hsp90-depleted cells

It has been reported that microtubule stability is essential for Golgi integrity and anterograde transport (Rogalski and Singer, 1984; Thyberg and Moskalewski, 1985; Ryan et al., 2012). Therefore, microtubule deacetylation and destabilization seemed to be causative for the defects of Golgi organization and vesicular transport in Hsp90-depleted cells. TSA was used to test this hypothesis.

**Figure 4:**
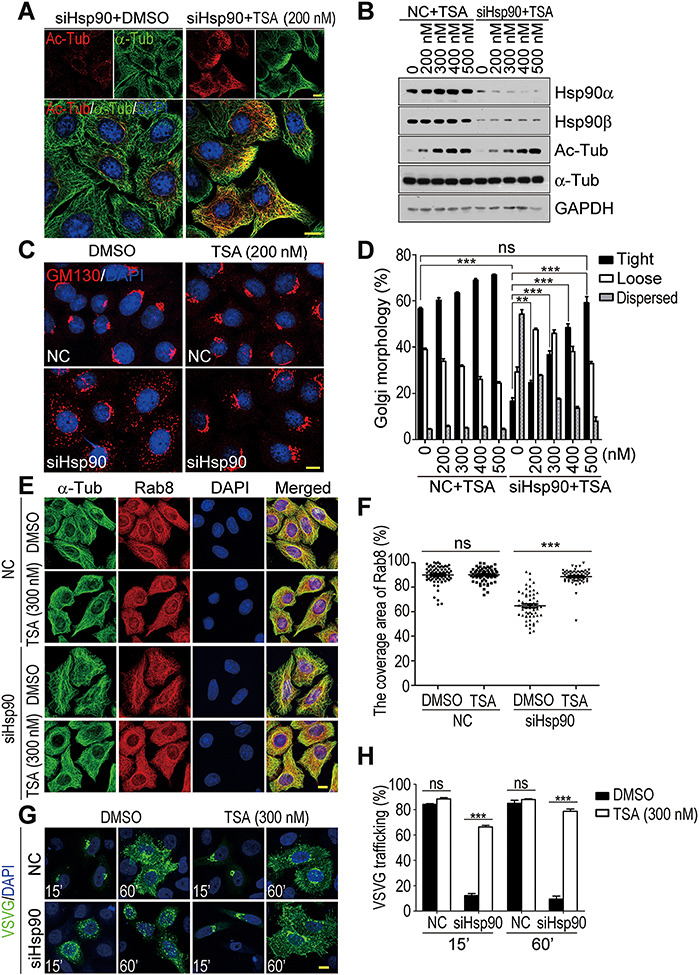
The restoration of Golgi organization and vesicular trafficking in Hsp90-depleted cells by TSA treatment. (**A** and **B**) The increase of microtubule acetylation in Hsp90-depleted HeLa cells treated with TSA at the indicated concentration (200, 300, 400, and 500 nM). NC, negative control siRNA; siHsp90, Hsp90 siRNA; DMSO, solvent control. (**C**) The restoration of Golgi organization in Hsp90-depleted cells by treating with TSA. The Golgi apparatus was revealed by immunofluorescence staining for GM130. (**D**) Quantification of the Golgi with various morphologies in TSA-treated cells. Golgi morphologies were categorized and counted as described in Figure 2B. The results were the average of three independent experiments, and at least 200 cells were analyzed per group for each experiment. Error bars represent SEM. Student’s *t*-test, ns, *P* > 0.05; **P < 0.01; ***P < 0.001. (**E**) The distribution of Rab8 in Hsp90-depleted cells with or without TSA treatment. α-Tub was stained to show the cell outline. (**F**) Quantification of cytoplasmic region with Rab8 in control or Hsp90-depleted cells after TSA treatment. Data are mean ± SEM of the results obtained from at least 60 cells for each group. Student’s *t*-test, ns, *P* > 0.05; ***P < 0.001. (**G**) The restoration of VSVG^tsO45^-GFP trafficking in Hsp90-depleted cells by TSA treatment. Control or Hsp90-depleted HeLa cells expressing VSVG^tsO45^-GFP were treated with vehicle DMSO or TSA and then shifted to 32°C to trace VSVG trafficking. (**H**) Quantification of VSVG^tsO45^-GFP trafficking in TSA-treated cells. VSVG^tsO45^-GFP reaching the tightly compacted Golgi (15′) and the PM (60′) was measured. The results were the average of three independent experiments, and at least 100 cells were analyzed for each experiment. Error bars represent SEM. Student’s *t*-test, ns, *P* > 0.05; ***P < 0.001. Scale bar, 10 μm.

TSA is a general inhibitor of class II histone deacetylases and reported to promote microtubule acetylation (Yoshida et al., 1990; Matsuyama et al., 2002; Zhang et al., 2015). Its treatment greatly increased microtubule acetylation in Hsp90-depleted cells (Figure 4A and B). In those TSA-treated cells, Hsp90 depletion-induced Golgi fragmentation was reversed (Figure 4C and D), Rab8 and microtubules became uniformly distributed (Figure 4E and F), and the impaired VSVG transport was also recovered (Figure 4G and H). By increasing microtubule acetylation, all the effects inhibited by Hsp90 depletion were restored to the normal state. Thus, microtubule destabilization was highly likely to be the primary cause for Golgi fragmentation and aberrant vesicular transport in Hsp90-depleted cells.

### Hsp90 interacts with and stabilizes MAP4 in HeLa cells

**Figure 5:**
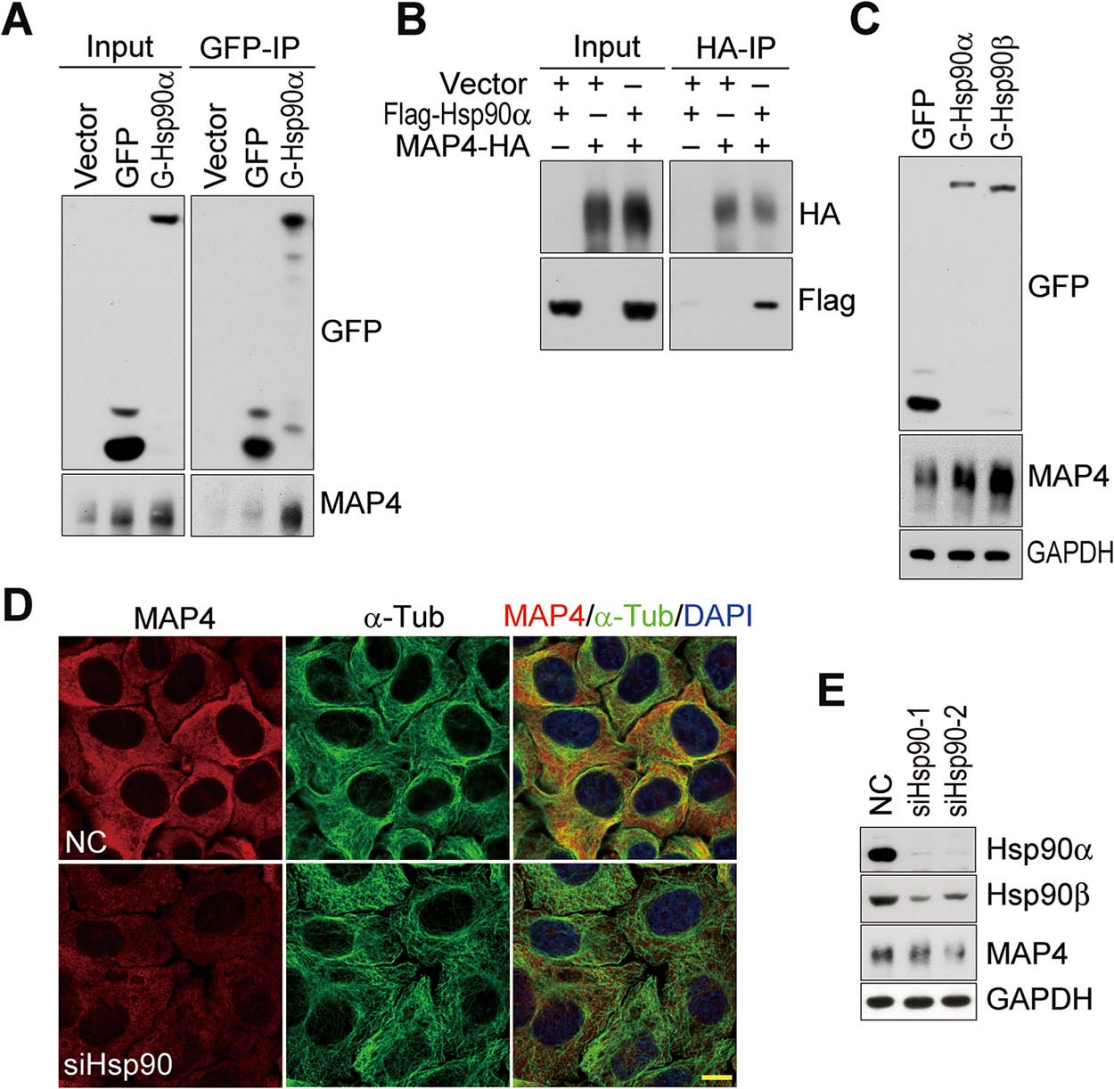
The identification of MAP4 as an Hsp90-interacting protein. (**A**) The interaction of MAP4 with Hsp90 *in vivo*. Stable HeLa cells expressing GFP or GFP-tagged Hsp90α were lysed and immunoprecipitated by anti-GFP affinity magnetic agarose. The immunoprecipitated samples were blotted for MAP4. (**B**) Interaction between exogenous Flag-tagged Hsp90α and HA-tagged MAP4 in HEK293T cells. Flag-tagged Hsp90α and HA-tagged MAP4 were co-expressed in HEK293T cells and MAP4 was immunoprecipitated by anti-HA affinity gel. The immunoprecipitated samples were detected for Flag-tagged Hsp90α by western blot. (**C**) Increase of MAP4 protein in Hsp90-overexpressed cells. HeLa cells stably expressing GFP or GFP-tagged Hsp90α or Hsp90β were lysed and equal amounts of proteins were loaded to detect MAP4 level by western bolt. GAPDH is the protein loading control. (**D** and **E**) Reduction of MAP4 in Hsp90-depleted HeLa cells detected by immunofluorescence (**D**) and western blot (**E**). Scale bar, 10 μm.

Microtubule deacetylation was the key event in Hsp90 depletion-induced Golgi fragmentation and vesicle trafficking blockade (Figure 4C and G). To identify the target protein by which Hsp90 affects microtubule acetylation, proteomic analysis was carried out and MAP4 was identified as an Hsp90-interacting protein. Their interaction was verified by co-immunoprecipitation (Figure 5A and B). MAP4 is a ubiquitously distributed microtubule-associated protein and functions by regulating the stability and dynamics of the microtubule (Nguyen et al., 1997; Kremer et al., 2005). In Hsp90α- or Hsp90β-overexpressed cells, the protein level of MAP4 was markedly increased (Figure 5C). In contrast, in Hsp90-depleted cells MAP4 protein was decreased (Figure 5D and E). The presence of Hsp90 was important for the stabilization of MAP4 in cells.

### MAP4 reduction causes microtubule destabilization, Golgi fragmentation, and defective vesicle trafficking in Hsp90-depleted cells

**Figure 6:**
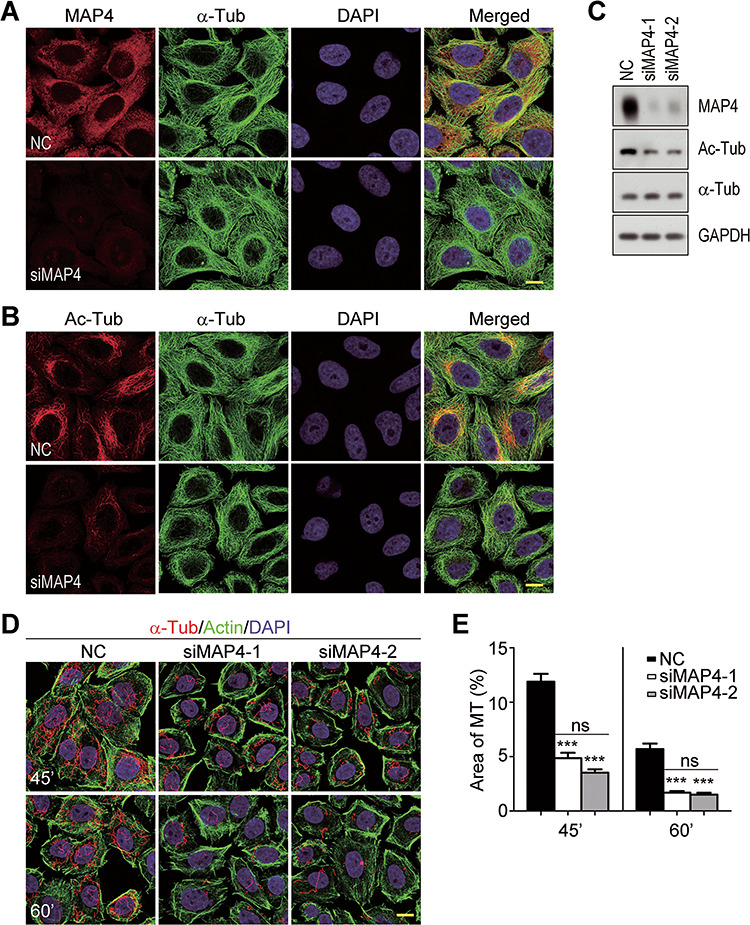
Microtubule destabilization induced by MAP4 depletion. (**A**) Knockdown of MAP4 by RNAi. The cells transfected with siRNAs were stained with antibody against MAP4. NC, negative control HeLa cells transfected with a scrambled siRNA sequence; siMAP4, HeLa cells transfected with siRNA sequences targeting MAP4. (**B**) Microtubule deacetylation induced by MAP4 depletion. The siRNA-treated cells were stained for α-Tub and Ac-Tub. (**C**) The reduction of MAP4 and α-tubulin acetylation in MAP4-depleted cells detected by western blot. (**D**) Microtubule destabilization induced by MAP4 depletion. Control (NC) and MAP4-depleted (siMAP4-1 and siMAP4-2) cells were treated with 2 μM nocodazole for 45 or 60 min (45′ or 60′). The remaining microtubules were detected by α-Tub staining. Actin was revealed by fluorescent-conjugated phalloidin to show cell outline. (**E**) Quantification of the remaining microtubules in control or MAP4-depleted cells after nocodazole treatment. Data are mean ± SEM of the results obtained from at least 60 cells per group. Student’s *t*-test, ns, *P* > 0.05; ***P < 0.001. Scale bar, 10 μm.

To verify that MAP4 was the target molecule of Hsp90 depletion to cause microtubule deacetylation and destabilization, MAP4 was knocked down by RNA interference. As shown in Figure 6A and C, two different siRNA sequences successfully decreased the protein level of MAP4. In these MAP4-depleted cells, α-tubulin acetylation was reduced (Figure 6B and C). Consequently, the microtubules were also destabilized as they were more susceptible to nocodazole treatment than those in control cells (Figure 6D and E). These results were consistent with the reports that MAP4 stabilizes microtubules (Nguyen et al., 1997; Kremer et al., 2005).

**Figure 7:**
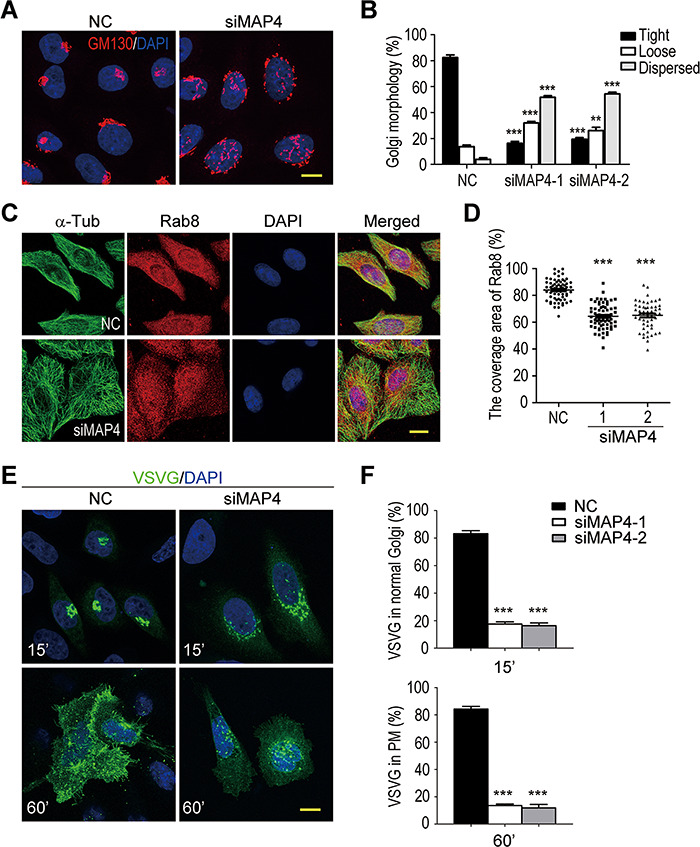
MAP4 depletion induces the same phenotypes as Hsp90 silencing in HeLa cells. (**A**) Golgi fragmentation in MAP4-depleted cells. Cells were stained with anti-GM130 antibody to reveal the Golgi apparatus. (**B**) Quantification of the Golgi with various morphologies in MAP4-depleted cells. Golgi morphologies were categorized and counted as described in Figure 2B. Data are mean ± SEM of the results obtained from three independent experiments, and at least 400 cells were counted per group for each experiment. Student’s *t*-test, ***P < 0.001. (**C**) Retention of Rab8 in perinuclear region of MAP4-depleted cells. α-Tub was stained to show the cell outline. (**D**) Quantification of cytoplasmic area with Rab8 in control or MAP4-depleted cells. The results were the ratio of Rab8-covered area relative to the cell outline area. Data are mean ± SEM of the results obtained from at least 52 cells for each group. Student’s *t*-test, ***P < 0.001. (**E**) Blocked VSVG^tsO45^-GFP trafficking in MAP4-depleted cells. Control or MAP4-depleted cells expressing VSVG^tsO45^-GFP were shifted from 40.5°C to 32°C. 15′ and 60′ indicate the incubation time at 32°C. (**F**) Quantification of VSVG^tsO45^-GFP localization in control and MAP4-depleted cells. Cells with VSVG^tsO45^-GFP in the tightly compacted Golgi (15′) or the PM (60′) were measured, and their percentages in VSVG^tsO45^-GFP-positive cell population were calculated. Data are mean ± SEM of the results obtained from three independent experiments, and at least 120 cells per group for each experiment. Student’s *t*-test, ***P < 0.001. Scale bar, 10 μm.

Besides the microtubule destabilization, MAP4 depletion also caused Golgi fragmentation and impaired anterograde VSVG^tsO45^-GFP transport (Figure 7A, B, E, and F). In these MAP4-depleted cells, Rab8 was more concentrated in the perinuclear region (Figure 7C and D). Also, TSA treatment could reverse the perinuclear distribution of Rab8 to a more uniformed cytoplasm localization, indicating microtubule deacetylation was a key factor in MAP4 depletion-induced cellular abnormalities (Supplementary Figure S2B and C). Overall, the phenotypes of MAP4-depleted cells were the same as those of Hsp90-silenced cells.

**Figure 8:**
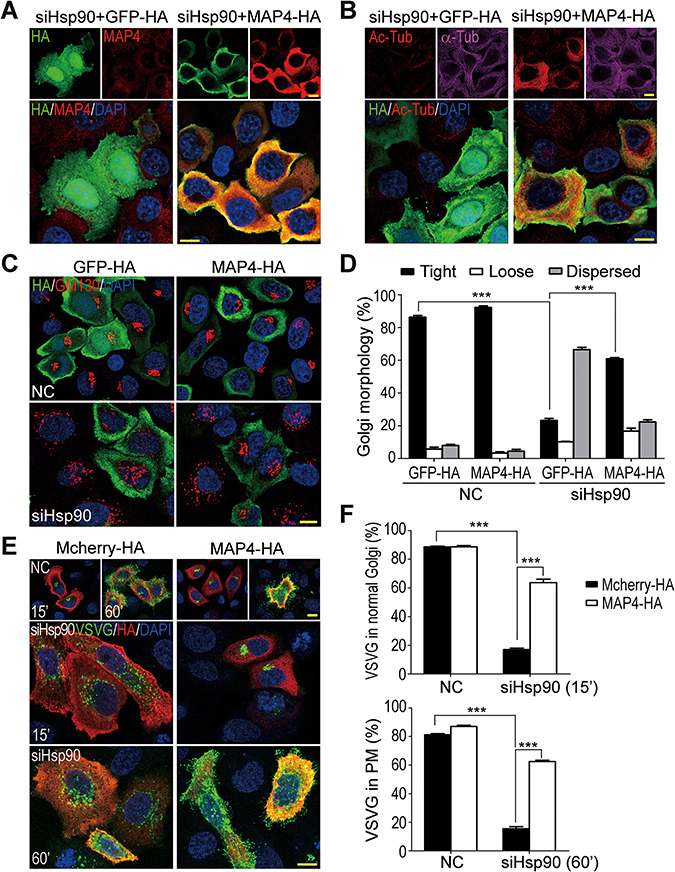
The restoration of Golgi organization and VSVG trafficking in Hsp90-depleted cells by MAP4 overexpression. (**A**) Exogenous expression of HA-tagged MAP4 in Hsp90-depleted HeLa cells. (**B**) Increased α-tubulin acetylation induced by exogenous expression of MAP4. Ac-Tub in GFP-HA or MAP4-HA transfected Hsp90-depleted HeLa cells was detected by immunofluorescence staining. (**C**) The restored Golgi organization in Hsp90-depleted cells by MAP4 overexpression. GFP-HA and MAP4-HA were transfected into Hsp90-depleted HeLa cells. GM130 was stained to reveal the Golgi apparatus. (**D**) Quantification of the Golgi with various morphologies in cells with MAP4 overexpression. The Golgi apparatus in tight, loose, or dispersed form as shown in Figure 2B was categorized and counted. The results were the average of three independent experiments, and at least 200 cells were analyzed for each experiment. Error bars represent SEM. Student’s *t*-test, ***P < 0.001. (**E**) The restoration of VSVG^tsO45^-GFP trafficking in Hsp90-depleted cells by MAP4 exogenous expression. Mcherry-HA or MAP4-HA and VSVG^tsO45^-GFP were co-transfected into Hsp90-depleted HeLa cells. VSVG trafficking was observed by shifting the cells from 40.5°C to 32°C. (**F**) Quantification of VSVG^tsO45^-GFP trafficking in cells with MAP4 overexpression. Cells with VSVG^tsO45^-GFP in the tightly compacted Golgi apparatus (15′) and the PM (60′) were counted. The results were the average of three independent experiments, and at least 110 cells were analyzed for each experiment. Error bars represent SEM. Student’s *t*-test, ***P < 0.001. Scale bar, 10 μm.

To further confirm that MAP4 is the target protein to mediate the effects of Hsp90 depletion, it was supplemented in Hsp90-depleted cells to rescue those affected cellular activities. As shown in Figure 8A and B, the expression of exogenous MAP4 in Hsp90-depleted cells increased the cellular MAP4 protein and promoted microtubule acetylation. It also rescued the fragmented Golgi and impaired anterograde vesicle transport (Figure 8C–F). Thus, MAP4 reduction was highly likely the main cause of microtubule deacetylation, Golgi disintegration, and vesicle trafficking defects in Hsp90-depleted cells.

## Discussion

Previously, we reported that the deficiency of Hsp90α, which is the major isoform of Hsp90 in retina, leads to photoreceptor degeneration and induces RP in mice (Wu et al., 2020). In photoreceptors, Hsp90α deficiency causes MAP1B degradation and α-tubulin deacetylation, accompanied by disintegrated Golgi and impaired rhodopsin transport. To establish that MAP1B degradation-induced microtubule destabilization is the causative factor for Hsp90α deficiency-induced membrane trafficking defects and photoreceptor degeneration, we depleted Hsp90 in HeLa cells to analyze its effects on microtubule stability, Golgi integrity, and vesicular trafficking. The results demonstrate that the effects of Hsp90 depletion in HeLa cells mimic the phenotypes of Hsp90α deficiency in photoreceptors (Figures 1–3). MAP1B is a neuron-specific microtubule-associated protein in nervous tissues (Hirokawa, 1994), and the ubiquitously distributed MAP4 is the major microtubule-associate protein in HeLa cells. In Hsp90-depleted HeLa cells, MAP4 is decreased and the supplement of exogenous MAP4 rescues all the phenotypes of Hsp90 depletion (Figures 5 and 8). Besides, the expression of exogenous MAP1B has the same effects as MAP4 to rescue the phenotypes induced by Hsp90 depletion in HeLa cells (Supplementary Figure S3). Furthermore, the phenotypes of Hsp90 depletion can also be induced by silencing MAP4 in HeLa cells (Figures 6 and 7). Taken together, we propose the sequences of the events induced by Hsp90 depletion: Hsp90 depletion leads to MAP4 reduction that causes α-tubulin deacetylation and microtubule destabilization, and then the destabilized microtubules cause Golgi disintegration and impair the vesicular transport from the Golgi to the PM. Based on these studies of Hsp90-depleted HeLa cells, it is clear that MAP1B degradation and microtubule destabilization causes photoreceptor degeneration and RP in Hsp90α-deficient mice (Wu et al., 2020). Thus, microtubule acetylation and stabilization could be a possible treatment for RP.

Hsp90 has been reported to colocalize with microtubules in both mitotic and interphase cells and interact with tubulin especially the acetylated form (Redmond et al., 1989; Garnier et al., 1998; Giustiniani et al., 2009). Hsp90 also interacts with tau to promote its association with microtubules, increase its solubility, and reduce its aggregation (Dou et al., 2003). We have made similar observations that Hsp90 clusters are colocalized with acetylated α-tubulin and GM130, and Hsp90 can interact with and regulate MAP1B and MAP4 (Figure 5; Supplementary Figure S4; Wu et al., 2020). All of these results indicate that Hsp90 is an important regulatory protein for the stability of microtubule-associated proteins as well as microtubules.

It has been well established that the organization, movement, and proper functions of cellular organelles are highly dependent on the cytoskeleton (Barr and Egerer, 2005; Starr, 2007; Bola and Allan, 2009). In the interphase, the Golgi is packed around the microtubule-organizing center (MTOC), where the stable microtubules such as acetylated or detyrosinated forms are highly abundant (Thyberg and Moskalewski, 1993). In the mitotic phase, the Golgi is disintegrated as microtubules undergo reorganization. Microtubule depolymerization or destabilization by nocodazole treatment or depletion of microtubule-associated proteins can lead to Golgi fragmentation and dispersion (Rogalski and Singer, 1984; Thyberg and Moskalewski, 1985; Ryan et al., 2012). The Golgi of the fibroblasts from two patients with MAP4 missense mutation is loosely compacted (Zahnleiter et al., 2015). The change of microtubule dynamics has broad consequences on cellular membrane dynamics and trafficking.

The disintegrated Golgi in Hsp90-depleted cells does not affect the membrane trafficking from the ER to the Golgi, but impairs the transport of membrane vesicles from the Golgi to the PM. VSVG^tsO45^-GFP can reach the Golgi apparatus in time when the temperature is shifted to 32°C, but it fails to leave the Golgi apparatus for the PM (Figures 1B and 2C). There is also a report that Hsp90 inhibitor treatment blocks VSVG transport from the Golgi to the PM in a cell-free system (Lotz et al., 2008). Our current study explains how the inhibition or depletion of Hsp90 eventually impairs the cellular vesicle trafficking system.

Most studies of Hsp90 focus on its function in signal transduction. Not much attention has been paid to its roles in maintaining the cytoskeletal system. Besides, the effects of cytoskeleton destabilization on cellular membrane trafficking indicate that Hsp90 has more functional implications on cellular activities than just the cytoskeleton maintenance. The involvement of membrane trafficking in the process of many functionally important proteins has further diversified the consequences of Hsp90-regulated cytoskeleton stabilization or destabilization. The function of Hsp90 in cytoskeletal regulation should be more emphasized in future studies.

## Materials and Methods

### Antibodies and reagents

For immunoblotting and immunofluorescence, antibodies against Hsp90α (ab74248, Abcam), Hsp90β (ab2927, Abcam), GFP (sc-9996, Santa Cruz Biotechnology), acetylated α-tubulin (sc-23950, Santa Cruz Biotechnology), α-tubulin (T5168, Sigma or MAB1864, Merck), GM130 (610822, BD Pharmingen), Rab8 (610844, BD Pharmingen), MAP4 (11229-1-AP, Proteintech), MAP1B (HPA022275, Sigma), Flag (F1804, Sigma), HA (H6908, Sigma), and GAPDH (G8795, Sigma) were used. Horseradish peroxidase-conjugated secondary antibodies, DAPI solution, and nocodazole were from Sigma. Alexa-Fluor 488-, 546-, or 647-conjugated secondary antibodies, anti-fade mounting medium, and Alexa Fluor 647 Phalloidin were from Molecular Probes/Invitrogen. TSA (S1045) was obtained from Selleck. Protease, phosphatase inhibitors, and Anti-Flag or HA affinity gel were from Biomake. Anti-GFP-magnetic agarose (D153-10) was from MBL.

### Cell culture and transfection

HeLa and HEK293T cells were cultured in DMEM medium containing 10% fetal bovine serum (FBS) at 37°C and 5% CO_2_.

For RNAi experiments, siRNAs, synthesized by GenePharma, were transfected into HeLa cells with Lipofectamine™ RNAiMAX Transfection Reagent (Invitrogen) following the protocol provided by the manufacturer. Cells were harvested for western blot or immunofluorescence 48 or 72 h after transfection. SiHsp90-1 was the siRNA sequence indiscriminately targeting to both Hsp90α and Hsp90β isoforms with 5′-GUUUGAGAACCUCUGCAAA-3′ as the sense strand and 5′-UUUGCAGAGGUUCUCAAAC-3′ as the antisense strand. SiHsp90-2 was the mixture of two siRNA sequences targeting Hsp90α and Hsp90β, respectively. The sequence targeting Hsp90α is sense 5′-GGAUCUGGUCAUCUUGCUU-3′, antisense 5′-AAGCAAGAUGACCAGAUCC-3′. The sequence targeting Hsp90β is sense 5′-GCUAGGUCUAGGUAUUGAU-3′, antisense 5′-AUCAAUACCUAGACCUAGC-3′. The sequences of siRNAs targeting MAP4 are: siMAP4-1 sense 5′-GAGUCAAAGAAGAAACCGU-3′, antisense 5′-ACGGUUUCUUCUUUGACUC-3′ (Seo et al., 2016); siMAP4-2 sense 5′-GAUAGUCCCAGCCAAGGAU-3′, antisense 5′-AUCCUUGGCUGGGACUAUC-3′ (Ghossoub et al., 2013). The siRNA of a scrambled sequence serving as negative control (NC) was offered by GenePharma, and the sequence is sense 5′-UUCUCCGAACGUGUCACGUTT-3′, antisense 5′-ACGUGACACGUUCGGAGAATT-3′.

For TSA treatment, HeLa cells were subjected to RNAi in the presence of TSA at the indicated concentration or vehicle DMSO. Cells were harvested for analysis after 72 h.

For exogenous MAP4 expression in Hsp90-depleted cells, 48 h after siRNA transfection HA-tagged MAP4 expression plasmid was transfected into these Hsp90-depleted or control cells with Lipofectamine 3000 Transfection Regent. Cells were harvested 24 h later.

### Analysis of VSVG^tsO45^-GFP/Mcherry trafficking

HeLa cells growing on coverslips were transfected with siRNAs. VSVG^tsO45^-GFP was introduced into cells with Lipofectamine 3000 Transfection Regent 46 h after siRNA transfection. After 6 h of incubation at 37°C, cells were shifted to 40.5°C for 20 h to accumulate VSVG^tsO45^-GFP in the ER. Anterograde transport of VSVG^tsO45^-GFP was analyzed after lowering the temperature to 32°C. At indicated time points, the cells were fixed with 4% paraformaldehyde (PFA) for immunofluorescence analysis.

To rule out the off-target effects, stable HeLa cell lines expressing siRNA-resistant GFP-tagged Hsp90α or Hsp90β mutant were constructed. These cells were then transfected with siRNAs targeting Hsp90 and VSVG^tsO45^-Mcherry plasmid for anterograde vesicle trafficking analysis following the procedures described in the preceding paragraph.

For TSA treatment, the inhibitor was added to the cell culture medium during the transfection of siRNAs. The transfection and analysis of VSVG^tsO45^-GFP trafficking were the same as those described in the previous paragraph.

To analyze the effects of MAP4 on VSVG^tsO45^ transport in Hsp90-depleted cells, MAP4-HA was co-transfected with VSVG^tsO45^-GFP into Hsp90-depleted cells.

### Immunofluorescence

Cells growing on glass coverslips were fixed with 4% PFA at room temperature for 30 min or methanol at −20°C for 5 min. After washing twice with PBS, the cells were incubated with Tris-buffered saline with Tween-20 (TBST) containing 0.1% Triton X-100 and 3% bovine serum albumin (BSA) for 30 min followed by primary antibody, fluorescein-conjugated secondary antibody, and DAPI solution. The coverslips were mounted with anti-fade mounting medium and observed under a Leica TCS SP8 confocal microscope.

### Quantification of Golgi morphology

Golgi morphologies were categorized into tight, loose, and dispersed forms. The tight form is the typical morphology of the Golgi with high compactness. Loose or dispersed Golgi was divided to distinguish the extent of Golgi fragmentation. The loose Golgi was at an early stage of fragmentation with loose and linear morphology and perinuclear localization. The dispersed Golgi was completely fragmented throughout the cell with punctate morphology. The cells were paraformaldehyde-fixed and stained for GM130 to reveal the Golgi. The cells were counted, and the rates of the Golgi with various morphologies were calculated.

### Measurement of microtubule stability

The measurement of microtubule stability was performed according to the reported protocols (Xu et al., 2017). Cells were treated with 2 μΜ nocodazole for 45 or 60 min and washed with PBS twice. After extracted with PBS containing 0.2% Triton X-100 and 2 μΜ nocodazole for 1 min, the cells were fixed with 4% paraformaldehyde for 30 min at room temperature. Microtubules and cell outline were revealed by anti-α-tubulin antibody and phalloidin-647 staining. The areas of microtubules and cell outline were measured with ImageJ (Fiji), and their ratio was calculated to represent the stability of microtubules.

### Measurement of cytoplasmic area with Rab8

The negative control and Hsp90-depleted HeLa cells were methanol-fixed and stained for Rab8 and α-tubulin. The cytoplasmic area covered by Rab8 and the cell outline area were measured with ImageJ (Fiji). Their ratio was calculated to measure the Rab8-covered cytoplasmic area.

### Protein interaction and immunoblotting

The endogenous interaction between Hsp90 and MAP4 was conducted by immunoprecipitation in stable HeLa cells expressing GFP-tagged Hsp90α. The interaction of exogenous Flag-tagged Hsp90α and HA-tagged MAP4 was conducted by transfecting HEK293T cells. Immunoprecipitation was carried out 48 h after transfection. The immunoprecipitated samples were analyzed by western blot (Qiu et al., 2001).

### Statistical analysis

Quantifications were acquired from three independent experiments and presented as mean ± SEM. Two tailed Student’s *t*-test was performed to calculate the *P*-value using GraphPad Prism Software. Differences were considered to be significant and marked as *P < 0.05, **P < 0.01, and ***P < 0.001.

## Supplementary Material

2019-0112_R2_Supplementary_Material_mjz093
