# Supplementary material for: The molecular chaperone Hsp90 maintains Golgi organization and vesicular trafficking by regulating microtubule stability
Source: J Mol Cell Biol. 2019 Sep 27;12(6):448–61. doi: 10.1093/jmcb/mjz093 (PMC7333477; doi:10.1093/jmcb/mjz093)
Supplement: 2019-0112_R2_Supplementary_Material_mjz093 [file 2019-0112_r2_supplementary_material_mjz093.pdf]

Supplementary Material

The molecular chaperone Hsp90 maintains Golgi organization and vesicular trafficking by regulating microtubule stability

Yuan Wu, Yubo Ding, Xiudan Zheng and Kan Liao*

Key Laboratory of Systems Biology, CAS Center for Excellence in Molecular Cell Science, Shanghai Institute of Biochemistry and Cell Biology, Chinese Academy of Sciences, University of Chinese Academy of Sciences, Shanghai 200031, China

*Correspondence should be addressed to: Kan Liao (kliao@sibs.ac.cn)

Contents

Supplementary Figures 3

Supplementary Figure S1. The morphological alternation of Golgi apparatus by Hsp90α or Hsp90β depletion 3

Supplementary Figure S2. The restoration of Rab8 localization in MAP4-depleted cells by trichostatin A treatment 4

Supplementary Figure S3. Increase of microtubule acetylation and restoration of Golgi organization in Hsp90-depleted cells by exogenous expression of MAP1B ... 5

Supplementary Figure S4. The localization of GFP-tagged Hsp90 in perinuclear cytoplasmic region and its colocalization with acetylated α-tubulin or GM130 6

Supplementary Figures:

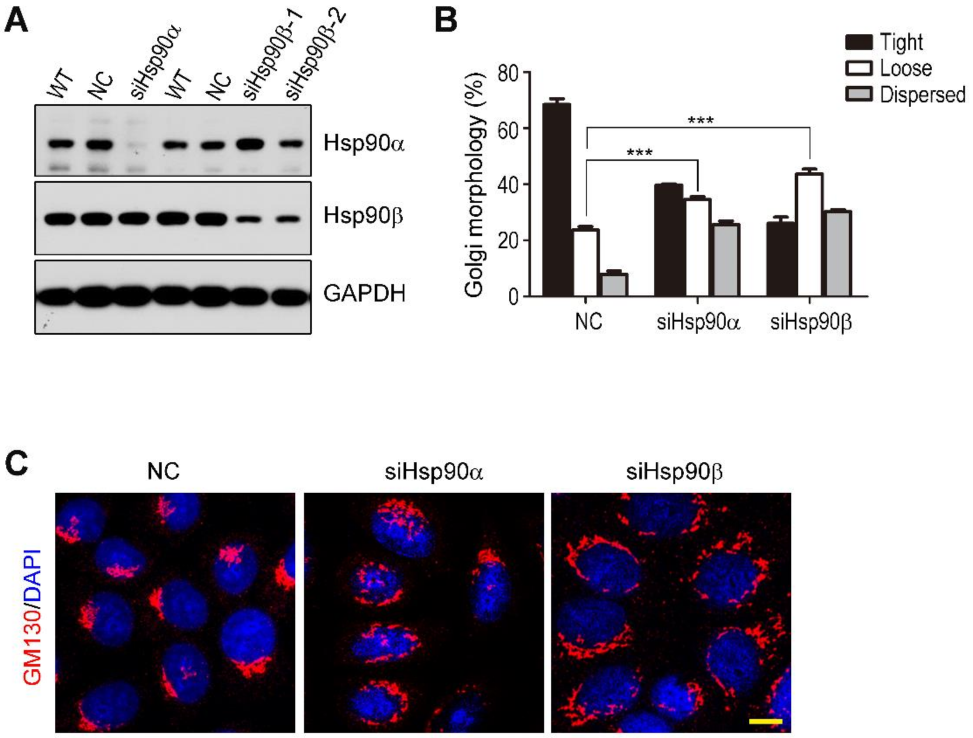

Supplementary Figure S1. The morphological alternation of Golgi apparatus by Hsp90α or Hsp90β depletion. A. Knockdown of Hsp90α or Hsp90β in HeLa cells by RNAi. *WT*, wild-type HeLa cells; *NC*, negative control cells transfected with a scrambled siRNA sequence; *siHsp90α,* HeLa cells transfected with a siRNA sequence targeting Hsp90α; *siHsp90β-1* and *siHsp90β-2*, HeLa cells transfected with two different siRNA sequences targeting Hsp90β. GAPDH is the protein loading control. **B.** Quantification of Golgi with various morphologies in control, Hsp90α- and Hsp90β-depleted cells. The results of control cells were the same as those in Fig. 2B, for the experiments were performed in the same batches. The results were the average of three independent experiments and at least 500 cells were analyzed per group for each experiment. Error bars represent SEM. Student's t-test, ** P<0.01; *** P<0.001. **C.** The loosely compacted Golgi in Hsp90α- and Hsp90β-depleted cells. Unlike the tightly compacted Golgi in most control cells, the Golgi was expanded and lack of compactness in the majority of Hsp90α- or Hsp90β-depleted cells. The bar is 10 μm.

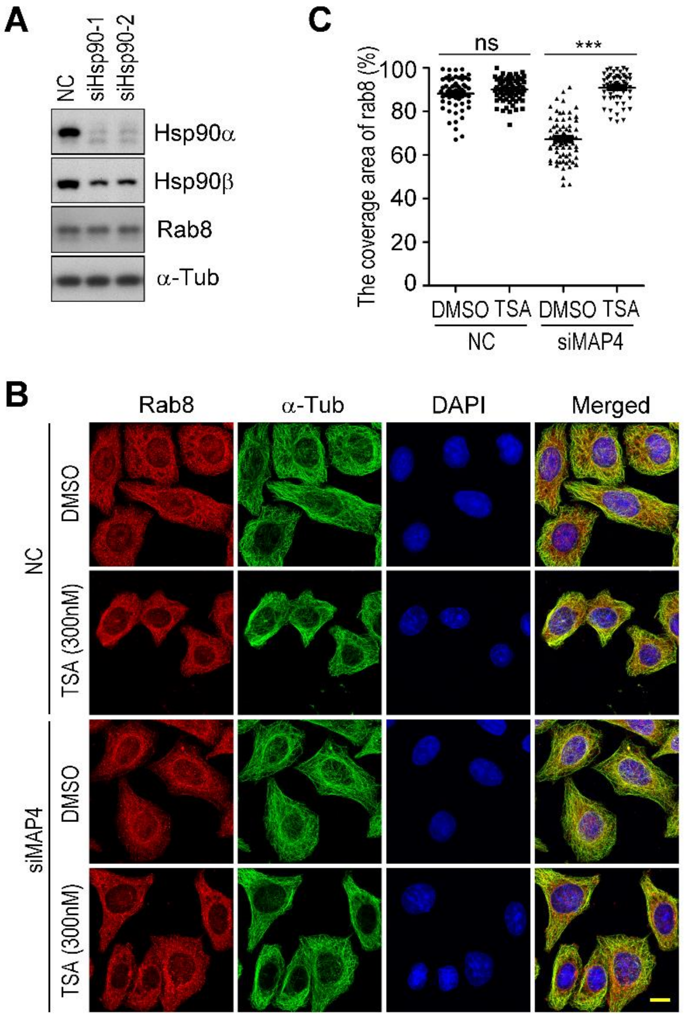

Supplementary Figure S2. The restoration of Rab8 localization in MAP4-depleted cells by trichostatin A treatment. The bar is 10 μm. **A.** Rab8 protein level in Hsp90-depleted cells. HeLa cells were transfected with a scrambled siRNA or Hsp90 siRNAs and then harvested for analysis. Equal amounts of proteins were loaded to perform Western blot. **B.** The distribution of Rab8 in MAP4-depleted cells with or without trichostatin A treatment. α-Tubulin was stained to show the cell outline. **C.** Quantification of cytoplasmic region with Rab8 in control or MAP4-depleted cells after trichostatin A treatment. Data are mean ± SEM of the results obtained from at least 58 cells for each group. Student's t-test, *ns* $P>0.05$; *** $P<0.001$.

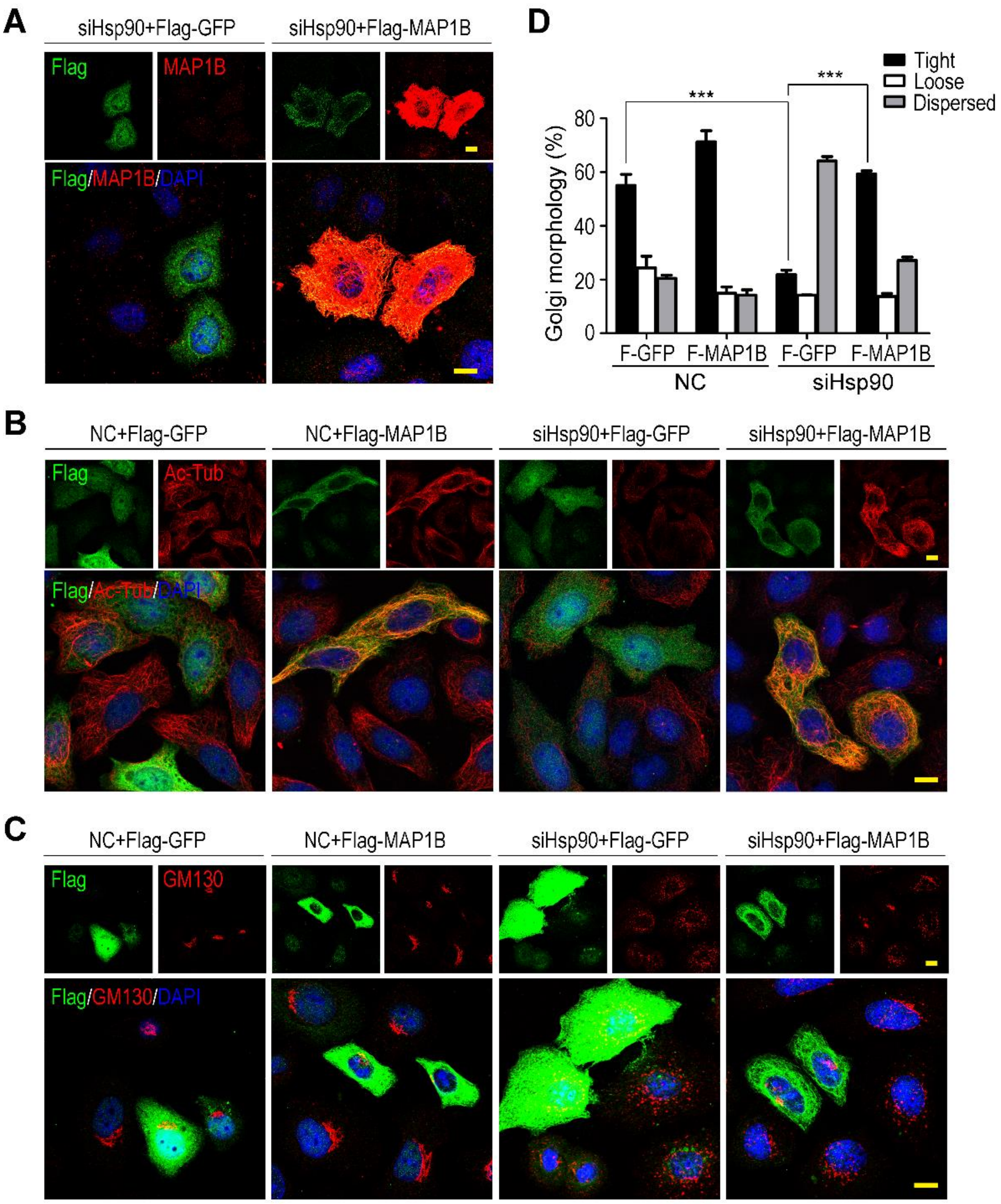

Supplementary Figure S3. Increase of microtubule acetylation and restoration of Golgi organization in Hsp90-depleted cells by exogenous expression of MAP1B. The bar is 10 μm. **A.** Overexpression of Flag-tagged MAP1B in Hsp90-depleted cells. The exogenous expression of MAP1B was validated by immunofluorescence staining for both Flag and MAP1B. MAP1B is a neuronal microtubule-associated protein and abundantly distributed in retinal photoreceptors. Little endogenous MAP1B exists in HeLa cells. **B.** The increase of α-tubulin acetylation by MAP1B overexpression. Flag-tagged GFP or MAP1B and acetylated α-tubulin were detected by immunofluorescence. **C.** The restoration of Golgi organization in Hsp90-depleted cells by MAP1B overexpression. GM130 was stained to reveal Golgi. **D.** Quantification of Golgi with various morphologies in siRNA-treated cells with MAP1B overexpression. The results were obtained from three independent experiments and at least 150 cells were scored in each experiment. Error bars represent SEM. Student's t test, *** $P<0.001$.

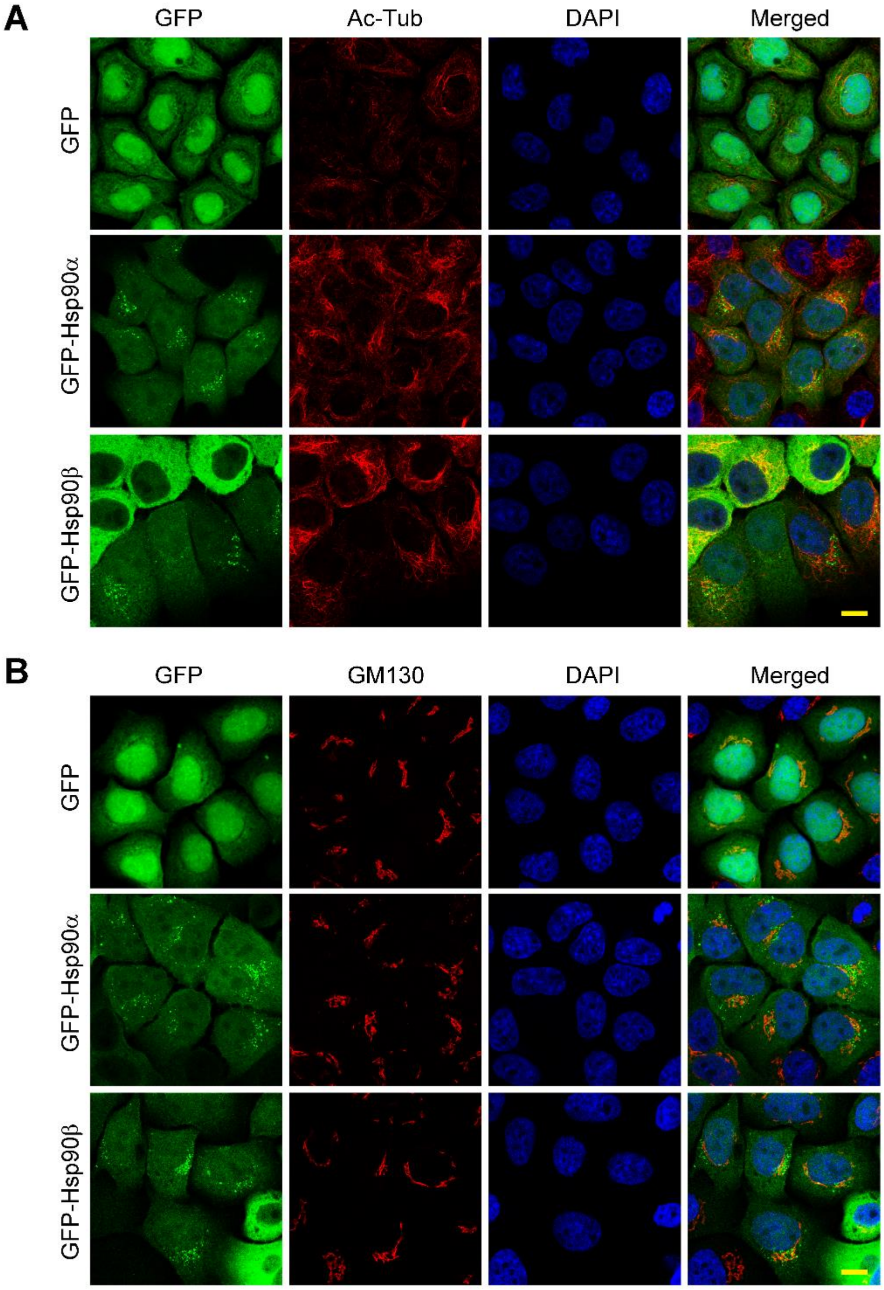

Supplementary Figure S4. The localization of GFP-tagged Hsp90 in perinuclear cytoplasmic region and its colocalization with acetylated α-tubulin or GM130. The bar is 10 μm. **A.** and **B.** Partial colocalization of Hsp90α or Hsp90β with acetylated α-tubulin (A) or GM130 (B). The cellular distribution of Hsp90 was obtained by tracing the GFP-tagged Hsp90 in stable HeLa cell lines. Acetylated α-tubulin or GM130 was revealed by immunofluorescence staining with respective antibody.
